# The potential of lactoferrin, ovotransferrin and lysozyme as antiviral and immune-modulating agents in COVID-19

**DOI:** 10.2217/fvl-2020-0170

**Published:** 2020-10-08

**Authors:** Jaclyn Kelly Mann, Thumbi Ndung'u

**Affiliations:** 1 ^1^HIV Pathogenesis Programme, University of KwaZulu-Natal, Durban 4001, South Africa; 2 ^2^Africa Health Research Institute, Durban, 4001, South Africa; 3 ^3^Ragon Institute of MGH, MIT & Harvard University, Cambridge, MA 02139, USA; 4 ^4^Max Planck Institute for Infection Biology, Chariteplatz, D-10117 Berlin, Germany; 5 ^5^Division of Infection & Immunity, University College London, London WC1E 6BT, UK

**Keywords:** COVID-19, glycerol, lactoferrin, lysozyme, novel coronavirus, ovotransferrin

## Abstract

Coronavirus disease 2019 (COVID-19), caused by SARS coronavirus 2 (SARS-CoV-2), is spreading rapidly with no established effective treatments. While most cases are mild, others experience uncontrolled inflammatory responses with oxidative stress, dysregulation of iron and coagulation as features. Lactoferrin, ovotransferrin and lysozyme are abundant, safe antimicrobials that have wide antiviral as well as immunomodulatory properties. In particular, lactoferrin restores iron homeostasis and inhibits replication of SARS-CoV, which is closely related to SARS-CoV-2. Ovotransferrin has antiviral peptides and activities that are shared with lactoferrin. Both lactoferrin and lysozyme are ‘immune sensing’ as they may stimulate immune responses or resolve inflammation. Mechanisms by which these antimicrobials may treat or prevent COVID-19, as well as sources and forms of these, are reviewed.

## Lay abstract

Severe coronavirus disease (COVID-19) is characterized by systemic inflammation, where unbound iron plays a significant role in tissue injury. There is no established effective treatment for COVID-19, and some treatment options being explored are unlikely to be widely available soon, especially in resource-limited settings. An abundant and safe antimicrobial that could act via oral ingestion to lower the risk of infection or prevent mild cases from progressing to severe disease would be ideal. Tear lactoferrin and lysozyme levels predict the risk of acquiring upper respiratory tract infections, and these antimicrobials are abundant in nature. Lactoferrin binds free iron and it has been shown to inhibit replication of the novel coronavirus. Ovotransferrin is closely related to lactoferrin and is more abundant (in hen egg white) – it shares iron-binding and antiviral properties of lactoferrin. Lactoferrin, ovotransferrin and lysozyme have several overlapping effects on microbes as well as the immune system, whereby they enhance or limit immune activation in a manner appropriate to the immune environment. Importantly, oral ingestion of these proteins in human and animal studies has shown safety as well as systemic effects, with the ability to limit immune pathology. Research into these products to prevent or treat severe COVID-19 disease is warranted.

A cluster of pneumonia cases of unknown etiology was first reported in December 2019 in Wuhan, China [1], and the disease, termed coronavirus disease 2019 (COVID-19), has subsequently spread rapidly throughout the world, posing a great threat to human health and the economy. The virus identified as the causative agent is the novel coronavirus (CoV) – SARS-CoV-2 [2] – which is highly related to the CoVs in bats and, of the human CoVs identified to date, is most similar (79.5% sequence identity) to SARS-CoV [3,4]. Currently, there are no proven effective treatments or vaccines for novel human CoVs [1], though several clinical trials of repurposed existing drugs and new vaccines are underway to test for efficacy in treating or preventing SARS-CoV-2 infection [5,6]. The aim of this review is to consider the potential of specific antimicrobial proteins that are abundant in nature to act as therapeutics in COVID-19 (as antivirals and/or counteracting the pathology) and to stimulate further research in this avenue.

## Pathology of COVID-19: the role of free iron & oxidative stress in tissue injury

Both SARS-CoV and SARS-CoV-2 use human ACE2 as the receptor for entry and have a similar overall binding mode [7]. SARS-CoV-2 is transmitted predominantly through contact with respiratory droplets from an infected individual [4]. Primary viral replication is thought to occur in the upper respiratory tract followed by replication in the lower respiratory tract and GI tract, and cells of the lung, heart, kidney and bladder may be infected. The infected individual may remain asymptomatic (estimated at 17.9% in one study [8]) or develop symptoms. Those who experience symptoms may have mild disease (80.9%), progress to severe disease (13.8%), require critical care (4.7%) or die (2.3% in all reported cases) [4]. Those with severe disease experience an uncontrolled inflammatory response (a cytokine storm) that may lead to acute respiratory distress syndrome (ARDS; characterized by widespread inflammation in the lung) as the typical manifestation or multi-organ failure through immune-induced damage [9], with ARDS occurring 7–14 days after symptom onset [10]. Manifestations are wide ranging, however, including gastrointestinal [11], renal [12], skeletal muscle [13] and neurological symptoms [14] for example.

Severe COVID-19 is reminiscent of hyperferritinemic syndrome (ferritin levels reaching thousands of units, leukopenia, abnormal liver function tests, severe hypercytokinemia and coagulopathy), under which septic shock and macrophage-activating syndrome (MAS) are also classified [15–17]. In particular, the cytokine profile in COVID-19 most closely resembles MAS, where ferritin directly activates macrophages to release pro-inflammatory cytokines (including IL-6 and TNF-α) and drives inflammation [16,18]. MAS is a type of secondary hemophagocytic lymphohistiocytosis, an under-recognized syndrome that may be triggered by viral infections and typically has pulmonary involvement (including ARDS) [19] – in this condition macrophages phagocytose red blood cells leading to anemia [20]. The role of macrophages in the excessive inflammation in COVID-19 is further supported by a recent article [21]. It is well documented that in conditions of systemic inflammation, oxidative stress mediates cell injury, and this is driven by increased free iron [15,22,23]. Further, there is disruption of iron homeostasis and increased free iron in the bronchoalveolar lavage fluid during ARDS and in other lung pathologies [24–26]. Free iron may directly react with oxygen to form superoxide radicals or with hydrogen peroxide (released by neutrophils and macrophages) to produce a highly toxic hydroxyl free radical [26]. Free radicals can also liberate iron from ferritin, the levels of which increase in response to sequester the reactive free iron as well as part of the acute phase response and due to leakage induced by immune damage [25,27], leading to further radical formation [27]. Serum ferritin levels are increased in the majority of COVID-19 patients [28], with markedly higher levels of serum ferritin in nonsurvivors compared with survivors [29]. Another consequence of increased free iron and reactive oxygen species, is the promotion of the development of advanced glycation end products (AGEs) [30], which play a central role in the pathogenesis of ARDS and other pulmonary inflammatory diseases [31,32]. Increased free iron also has direct effects on fibrinogen, fibrin and erythrocyte morphology and promotes a procoagulant state [22]. Intravascular coagulation is a frequent finding in the more severe COVID-19 cases, with D-dimer levels strongly linked to disease severity [33]. D-dimer levels rise prior to IL-6, and are not therefore simply secondary to systemic inflammation. Contributing to oxidative stress, and further immune activation, is increased angiotensin II (a product of the inflammation-driven activation of the renin–angiotensin system [RAS] [34]), which is potentiated by the binding of SARS-CoV-2 to the ACE2 receptor and is linearly associated with viral load and lung injury in COVID-19 [35]. In summary, systemic inflammation with associated oxidative stress, dysregulation of iron metabolism and coagulation are key features of COVID-19.

## Lactoferrin, ovotransferrin & lysozyme as potential therapeutics in COVID-19

A vaccine for SARS-CoV-2 is expected to be ready at minimum in 12–18 months from now. An abundantly available antimicrobial which could lower the risk of infection or prevent mild disease from becoming severe disease (where progression to this stage occurs in about 20% of symptomatic individuals and takes ~1–2 weeks after mild symptoms are experienced) would have great value. Drugs already approved for other uses are currently being tested in clinical trials; however, there is currently little evidence to show that they are having much effect [5]. While remdesivir marginally shortens recovery time in hospitalized patients [6], it is unlikely to be widely available soon, especially in resource-limited settings.

**Figure 1.**
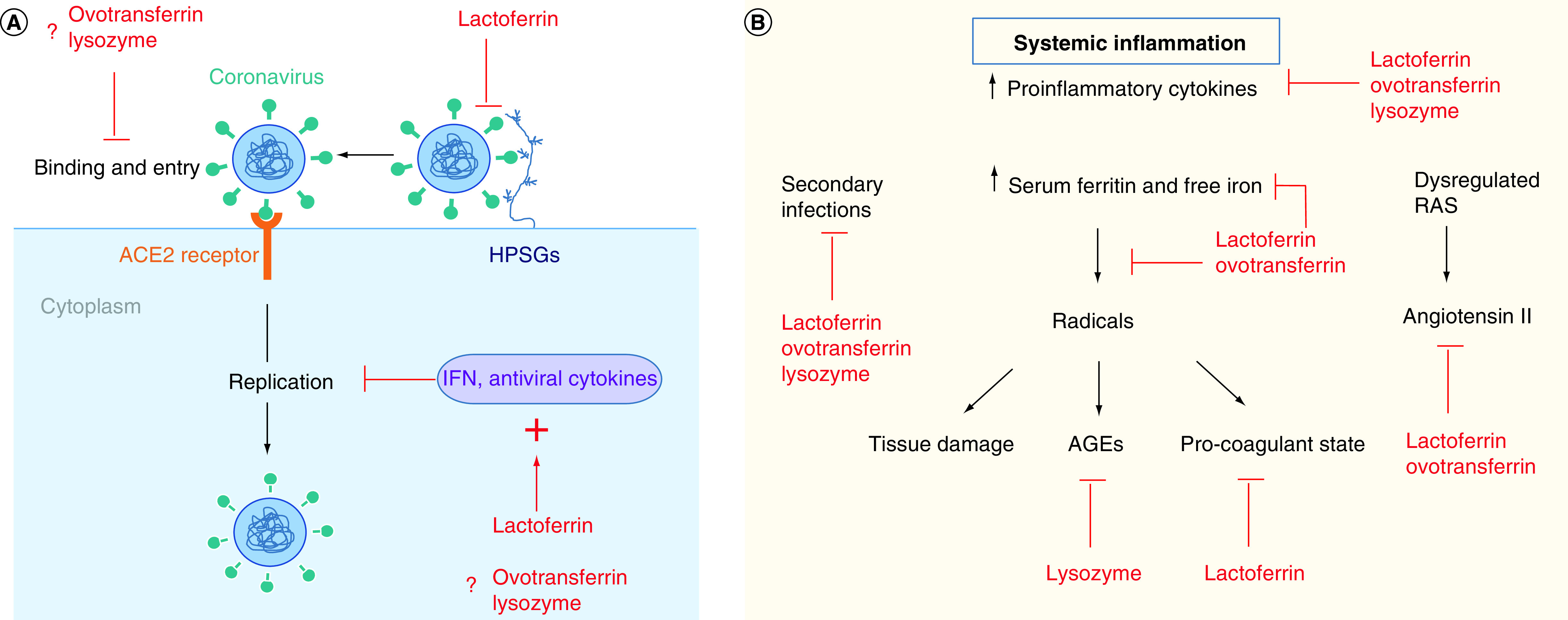
Potential benefits of lactoferrin, ovotransferrin & lysozyme in SARS coronavirus 2 infection. Potential antiviral **(A)** and immunomodulatory **(B)** effects of these proteins in SARS-CoV-2 infection are illustrated. The mechanisms are further detailed in Table 1. The antiviral effects of lactoferrin against SARS-CoV-2 replication have been demonstrated. The antiviral mechanisms of ovotransferrin and lysozyme are inferred from their known effects on other viruses, however their effects against SARS-CoV-2 are currently unknown. AGE: Advanced glycation end product; CoV: Coronavirus; HSPG: Heparan sulphate proteoglycan; RAS: Renin–angiotensin system.

Recently it was reported that tear lactoferrin and lysozyme are relevant biomarkers of mucosal immune competence and that the levels of these predict the risk of acquiring upper respiratory tract infections [36]. Lactoferrin and lysozyme concentrations decrease with age [37,38], potentially increasing risk for respiratory infections. Lactoferrin and lysozyme are among the most abundant antimicrobials found in nature that are widely distributed in animal tissues and secretions [39–41], and are considered among the most promising antimicrobials to become medicines for clinical use [42,43]. They both act widely against bacteria, viruses and fungi, as well as having positive stimulatory effects on the immune system yet dampening the pathological effects of an overreacting immune system. Lactoferrin and lysozyme are found in markedly high concentrations in tears compared with any other body fluid, and lactoferrin is found in similarly high concentration in breast milk and colostrum – this indicates the important role of these proteins in defense [39,44,45]. However, the usual concentrations are only just adequate, and lower than normal levels in these secretions increase susceptibility to infection [44]. While SARS-CoV-2 is readily detected in throat swabs, nasal swabs, saliva and sputum, and in a third of patients in feces [46–48], the virus is only infrequently detected in tears in a similar timeframe [49–51]. When detected in tears, this has been in patients that had conjunctivitis symptoms [49,51]. A similar scenario is reported for SARS-CoV [52,53]. The potential activity of lactoferrin and lysozyme against SARS-CoV-2 and against the immune-mediated pathology in COVID-19 (summarized in Figure 1 and Table 1) is considered. Since ovotransferrin is more abundantly available than lactoferrin and can substitute lactoferrin in many applications [54], its potential as a COVID-19 therapeutic is also reviewed.

**Table 1.** The overlapping antimicrobial and immunomodulatory effects of lactoferrin, ovotransferrin and lysozyme.

Protein	Form	Effect	Mechanisms	Ref.
**Lactoferrin**	Intact and/or peptides	Antiviral^†^	Interaction with virus surface, DNA or cell surfaces required for virus entry and induction of type I interferons	[39,55–58]
	Intact and peptides	Antibacterial	Iron sequestration Direct interaction with bacterial surface	[39,59–61]
	Intact and peptides	Antifungal	Iron sequestration Direct interaction with fungal surface	[62]
	Intact	Immune enhancement, immune suppression reversal^‡^	Enhances natural killer cell activity Enhances T-cell responses (helper and cytotoxic T-cell responses) Elevation of antibody response	[63–65]
	Intact and/or peptides	Anti-inflammatory^‡^	Suppresses extracellular traps released by neutrophils Polarizes macrophages to M2 type Downregulates IL-6 and TNF-α in those with high immune activation Binds to angiotensin II receptor type 1 to inhibit angiotensin II pro-inflammatory activity	[43,66–69]
	Intact and large fragments	Iron homeostasis*	Sequestering free iron Restoring levels of iron-binding proteins	[70]
	Intact and/or large fragments	Antioxidant*	Sequestering free iron Reduces intracellular levels of reactive oxygen species Increases antioxidant capacity of serum	[63,64]
	Intact and peptides	Antithrombotic	Through sequestering free iron (free iron induces procoagulant state) Inhibits platelet aggregation	[71]
**Ovotransferrin**	Intact and peptides	Antiviral	Upregulation of type 1 interferon in virus-infected macrophages Restriction of virus entry Antiviral peptides that have sequence homology with antiviral lactoferrin peptides	[72,73]
	Intact and peptides	Antibacterial	Iron sequestration Direct interaction with bacterial surface	[54,74]
	Intact and peptides	Antifungal	Iron sequestration Direct interaction with fungal surface	[75,76]
	Intact	Immune enhancement, immune restoration in immunosuppression model	Enhanced phagocytic activity as well as cytokine production of macrophages Enhanced intestinal immune responses: dendritic cell maturation, Th1/Th2 balance restored and humoral immunity promoted	[77,78]
	Peptides	Anti-inflammatory	Downregulates IL-6 and TNF-α and myeloperoxidase activity in peritonitis Binds to angiotensin II receptor type 1 to inhibit angiotensin II pro-inflammatory activity ACE inhibitory activity (antihypertensive)	[79–82]
	Intact	Iron-binding activity*	Sequestering free iron	[83]
	Intact and peptides	Antioxidant*	Sequestering free iron Free radical scavenging	[79,84]
**Lysozyme**	Intact and peptides	Antiviral	Inhibits viral entry by binding to cell receptors or virus – cationic and hydrophobic nature is required rather than enzymatic activity Binds nucleic acids Inhibits virus-induced cell fusion Affects cell signaling, including NF-κB pathway, to influence susceptibility to infection	[85–88]
	Intact and/or peptides	Antibacterial	Hydrolyzes cell wall of gram-positive bacteria (enzyme activity) Insert into and form pores in negatively charged bacterial membranes	[40]
	Intact and/or peptides	Antifungal	Enzymatic activity Cationic nature leading to membrane destabilization Agglutination effect	[89–91]
	Intact and/or peptides	Enhance or limit immune responses^‡^	Lysozyme in bacteria-containing phagosomes activates the pro-inflammatory responses of neutrophils and macrophages Decreases chemotaxis in neutrophils Suppresses TNF-α and IL-6 production by macrophages Facilitates excretion of AGEs Disrupts binding of peptidoglycans to complement ACE inhibitory activity Antioxidant activity	[30,40,92–94]

† Specific anticoronavirus activity has been demonstrated: inhibits SARS-CoV cell entry by binding to HSPGs; inhibits entry and postentry steps of SARS-CoV-2 replication and elevates interferon-stimulated genes in SARS-CoV-2-infected cells.

‡ The action is of immune homeostasis – its action is appropriate in the context of the immune environment [67].

*Iron sequestration has an antioxidant effect, and, in turn, these activities of lactoferrin/ovotransferrin have an anti-inflammatory effect – they limit immune pathology.

ACE: Angiotensin-converting enzyme; AGE: Advanced glycation end product; AT1: AT1 is angiotensin II receptor type 1; CoV: Coronavirus; HSPG: Heparan sulphate proteoglycan; M2: Anti-inflammatory macrophages; SARS-CoV: Severe acute respiratory syndrome coronavirus.

## Lactoferrin as an antiviral & immune modulator

Lactoferrin sequesters free iron, removing a substrate required for bacterial growth; however, it also has antimicrobial effects independent of iron sequestration [39]. Lactoferrin is cationic (highly positively charged) and this enables interaction with various negatively charged microbial and viral surfaces, DNA, as well as with cell surfaces that are required for bacterial and viral adhesion or for early interactions required for viral entry [39]. Lactoferrin may also exert antiviral effects intracellularly [55]. Potent antiviral effects of both human and bovine lactoferrin have been shown against both enveloped and naked viruses such as cytomegalovirus, herpes simplex virus and hepatitis B and C virus among others, whether in the metal saturated or apo form [39,66]. Bovine lactoferrin may have higher antiviral activity than human lactoferrin – they are highly similar and possess identical multifunctions [43]. Therefore, bovine lactoferrin is a good equivalent for human lactoferrin, especially since it is recognized by the European Food Safety Authority as a safe dietary supplement with medicinal properties and no contraindications [95]. Importantly, bovine lactoferrin inhibits SARS-CoV cell entry by binding to heparan sulphate proteoglycans (HSPGs) [56]. HSPGs on the cell surface provide an anchoring site on the cell surface and many viruses, including SARS-CoV, employ HSPGs for adhesion to susceptible cells. SARS-CoV-2 entry is highly similar to that of SARS-CoV [7] and was recently shown to be susceptible to lactoferrin-mediated inhibition of entry [57]. Lactoferrin inhibited both entry and postentry steps of SARS-CoV-2 replication, and elevated interferon-stimulated genes [57].

Besides its direct antimicrobial effect, through sequestering free iron and restoring iron homeostasis, lactoferrin reduces oxidative stress and inflammation, which is pertinent to the COVID-19 pathology. Lactoferrin counteracts iron dysregulation through sequestering free iron and restoring levels of various proteins (ferroportin, ceruloplasmin, transferrin receptor 1 and ferritin) that are altered during inflammation [70,96]. Lactoferrin reduces intracellular levels of reactive oxygen species as well as reducing oxidative stress-induced apoptosis [63], and short-term oral administration of bovine lactoferrin improves antioxidant capacity [64]. Importantly, lactoferrin can ‘sense’ the immune activation status and respond accordingly [67]. For example, in individuals with high baseline immune activation bovine lactoferrin downregulates IL-6 and TNF-α production by peripheral blood mononuclear cells (after 7 days of 40 mg per day oral administration) while in those with low immune activation, bovine lactoferrin upregulated these cytokines [67]. Lactoferrin suppresses extracellular traps released by neutrophils during inflammation [68], and has also been shown to stimulate pro-inflammatory macrophages (M1) to change to the anti-inflammatory macrophages (M2) type [43,66]. Similarly, pasteurized whole cow’s milk has been shown to polarize macrophages from M1 toward a proresolving M2 phenotype [97]. In addition, lactoferrin-derived peptides inhibit angiotensin II pro-inflammatory activity through binding to the angiotensin II receptor type 1 [69], and lactoferrin as well as other peptides in cow’s milk have an antithrombotic effect [71]. These effects of counteracting iron dysregulation, oxidative stress, neutrophil and macrophage-induced inflammation, RAS-induced inflammation and thrombosis are highly relevant to COVID-19. Further, lactoferrin shows potential benefit in Alzheimer’s disease through decreasing amyloid-beta aggregation (which leads to inflammation and neuron degeneration) [43,98]. This aggregation may be induced by microbes and this has been suggested for SARS-CoV-2 [99] – a potential neuroprotective role of lactoferrin in COVID-19 is a hypothesis requiring further investigation.

Oral administration of lactoferrin, usually bovine lactoferrin, in human and animal studies of various inflammatory disease states shows safety [43]. In animal studies, oral bovine lactoferrin was shown to decrease inflammation and myeloperoxidase (a marker of neutrophil infiltration) in inflammatory bowel disease [43,100]. In animal models of sepsis, a single oral dose of lactoferrin prior to insult protected against progression of insult-induced systemic inflammatory responses [63] and when orally administered after sepsis-induced lung injury, bovine lactoferrin was an effective therapeutic [101]. Further showing positive effects of lactoferrin in lung pathology, oral doses of human or mouse lactoferrin reduced *Mycobacterium tuberculosis*-induced lung pathology in a mouse model [102] and aerosolized bovine lactoferrin administered in a mouse model of cystic fibrosis with a *Pseudomonas aeruginosa* lung infection resulted in decreased bacterial load, decreased infiltrated leukocytes and reduced iron overload [70].

In human studies, lactoferrin decreased late onset sepsis and necrotizing enterocolitis in preterm infants [103], and oral bovine lactoferrin (250 mg/day for 3 months) decreased serum IL-6 and increased IL-10 as well as improved antioxidant activity in Alzheimer’s disease [43,104]. In pregnant women suffering from anemia and/or thrombophilia, 100 mg of bovine lactoferrin taken orally twice a day improved hematological parameters, including serum iron, serum ferritin, hemoglobin and IL-6 levels, more effectively than the standard of care [105]. Clinical effect has also been observed following lactoferrin administration in viral diseases. In hepatitis C-infected patients who responded to bovine lactoferrin monotherapy, when bovine lactoferrin was then combined with ribavirin and interferon, there was a sustained virologic response in 55% of individuals compared with a sustained virologic response in 18% of individuals who were treated with a combination of ribavirin and interferon alone [106]. Long-term oral consumption of bovine lactoferrin-containing products including yoghurt and milk (in the range of 100 to 500 mg lactoferrin per day) either reduces incidence or ameliorates symptoms of common viral infections, such as norovirus, likely though direct antiviral activity as well as the enhancement of systemic immunity (increased natural killer cell activity and Th1 cytokine responses) achieved by bovine lactoferrin consumption [107]. Importantly, Serrano *et al.*, reported that a liposomal bovine lactoferrin nutritional syrup administered at 256–384 mg lactoferrin/day resolved symptoms of COVID-19 patients within 4–5 days and considering their 256 contacts who received half this daily dose, none developed symptoms of the infection [95].

## Ovotransferrin as an antiviral & immune modulator

Ovotransferrin shares many of the same activities as human/bovine lactoferrin and is more abundant than the latter [54]. Ovotransferrin combines the iron transport and defense functions of mammalian serum transferrin and lactoferrin, respectively, and shares about 50% sequence homology with each protein [83]. However, the structural analogy between ovotransferrin and lactoferrin is much closer than the sequence homology [108] and similar clusters of positively charged residues responsible for antiviral activity are found in the N-lobes of these proteins [72].

Ovotransferrin not only has antifungal activity [75] and a wide range of antibacterial activity through sequestration of iron and through binding to bacterial surfaces via cationic peptides [54,74], but the antiviral activity of intact ovotransferrin was greater than that of intact bovine lactoferrin when studying Marek’s disease virus [72]. Peptides in ovotransferrin that have high sequence homology with these bovine lactoferrin and human lactoferrin peptides acting against herpes simplex virus, human cytomegalovirus and adenovirus, were shown to have double the antiviral activity compared with the bovine lactoferrin peptides [72]. Recently, it was also reported that ovotransferrin upregulates antiviral interferon I in virus-infected macrophages [73].

Ovotransferrin has immunomodulatory, antioxidant and anti-inflammatory properties, and due to these properties it is being investigated as a therapeutic for cancer and cardiovascular disease [109,110]. Ovotransferrin, as well as hydrolyzates, are able to scavenge free radicals, with higher activity than other known antioxidants such as ascorbate (vitamin C) – ovotransferrin showed protective effects against oxidative stress-induced DNA damage, that was occurring via reaction of iron with hydrogen peroxide, in human leukocytes [79,84]. Specifically, 16 antioxidant peptides are derived from egg white hydrolyzate, where ovotransferrin peptides are in one of the most active fractions [111]. An ovotransferrin peptide attenuates TNF-α-induced inflammation and superoxide generation in endothelial cells [112]. Hydrolyzates of ovotransferrin, as well as other egg white peptides, have also shown potent ACE inhibitory activity [79,80] and as is the case for bovine lactoferrin, an ovotransferrin peptide blocked angiotensin II effects via the angiotensin II receptor type 1 [81], thereby reducing inflammation potentiated by RAS activation. In an animal model study of peritonitis, ingestion of 40 mg/kg feed of an ovotransferrin peptide significantly attenuated the inflammatory responses: serum levels of TNF-α, IL-6 and myeloperoxidase activity were significantly reduced [82]. As is the case for bovine lactoferrin, these described activities of ovotransferrin, are highly pertinent to COVID-19 pathology.

## Lysozyme as an antiviral & immune modulator

Lysozyme kills gram-positive bacteria through hydrolyzing the β-1,4 glycosidic bond between *N*-acetylglucosamine and *N*-acetylmuramic acid in the bacterial cell wall [40]. However, besides its enzymatic activity, it exerts antimicrobial effects through its cationic nature which enables it to bind to negatively charged surfaces (as in the case of lactoferrin), thereby expanding its activity well beyond that of gram-positive bacteria [40,41,89]. The immunomodulatory function of lysozyme has only recently been appreciated [40]. Although lysozyme acting on microbes within neutrophils and macrophages increases their proinflammatory response, when it is released extracellularly by these cells as well as epithelial cells, it limits inflammation: it decreases the oxidative burst and chemotaxis in neutrophils [92], it significantly suppresses TNF-α and IL-6 production by macrophages [93], it binds and decreases circulating levels of AGEs (which are pro-oxidative) as well as enhancing their renal excretion [30], and exogenous lysozyme disrupts the ability of peptidoglycan to bind complement factors that act as anaphylotoxins [40]. Furthermore, when subjected to simulated gastrointestinal digestion, the hydrolyzate of hen egg white lysozyme (HEWL) showed marked antioxidant and ACE-inhibitory activity [94]. As described earlier, the oxidative stress (including involvement of AGEs), inflammation induced by neutrophils and macrophages, the TNF-α and IL-6 cytokines, and an activated RAS system are features in ARDS and/or severe COVID-19. It is noteworthy that, as is the case for lactoferrin, lysozyme has a neuroprotective function in Alzheimer’s disease through preventing amyloid-beta aggregation [113], which may have implications for the neurological manifestations in severe COVID-19.

From mouse and porcine models it is clear that lysozyme plays an important role in limiting inflammation systemically, resulting in decreased immune-driven pathology [40,114,115]. Human clinical trials with lysozyme are limited [41,116], but have shown, mostly through oral administration of HEWL, antiviral effects against herpes (through oral administration of HEWL at 1 g/day [41,117]), measles [116,118] and hepatitis (60–170 mg/day of lysozyme chloride for 4–24 weeks significantly reduced post-transfusion hepatitis incidence to 8% compared with 20% [119]), successful treatment of gum infections (750 mg/day [41,120]) and skin ulcers [116,121], improvement of immune responses in cancer patients with immune suppression [41], and rapid resolution of inflammatory foci and stabilization of lysozyme levels in serum and stool of premature infants with diseases following 50 mg/l supplementation in milk for 2–3 weeks [116,122]. Human lysozyme in combination with bovine lactoferrin (0.2 g lysozyme and 1.5 g bovine lactoferrin per day) reduced enteric dysfunction in Malawian children [123]. No local or systemic unfavorable effects have been reported in these human trials.

Regarding the investigation of lysozyme in lung diseases, in Eastern Europe, HEWL has been used successfully in combination with antibiotics to treat bronchitis and pneumonia in humans with no respiratory or systemic toxicity [124,125]. Administration of lysozyme through aerosols to treat pneumonia has been investigated in animal models [126,127]. A 1% solution of aerosolized human lysozyme in hamsters with *P. aeruginosa*-induced pneumonia resulted in decreased lung histopathological changes, alveolar septal apoptosis, neutrophils and other leukocytes in the bronchiolar lavage fluid as well as increased activity of lysozyme in that fluid [126]. However, it should be noted that lysozyme impairs the ability of hyaluronan to prevent elastase injury to elastic fibers through binding of the lysozyme to the elastic fibers, and thus on inhalation of lysozyme in an animal model of emphysema, airspaces further increased [128], which cautions against the inhalation route of administration of lysozyme in similar disease states.

## Sources, forms & practical use of lactoferrin, ovotransferrin & lysozyme

Cow’s milk is the most readily available source of lactoferrin, with an average concentration of 0.174 g/l in low heat pasteurized cow’s milk (and 1.2 g/kg in semihard cheese produced from that milk) [129], which is in good agreement with other studies [130–132], though the range experienced (0.03–0.486 g/l) is dependent on several factors [133]. The concentration in colostrum is higher, but varies greatly between breeds and may be anything between 0.3 and 5 g/l, and is typically at the lower end of the range [134–136]. The degradation of bovine lactoferrin in milk with low heat pasteurization (72°C for 5 s) is minimal [129,137], while ultrahigh temperature (UHT) processes significantly denature the protein [138]. Large-scale isolation of bovine lactoferrin is performed from cheese whey [139] where only 19% of the total bovine lactoferrin in milk is found [129], and the cost of purified bovine lactoferrin remains high – hence methods to achieve large-scale production of lactoferrin are being developed [140,141]. Bovine lactoferrin is sold in bulk powder form, capsules (typically 250–300 mg), liposomal syrups (32 mg/10 ml) or as a liposomal lactoferrin nebulizer [95,123,142]. The majority of bovine lactoferrin taken orally can be considered to survive gastric transit (62% for the apo form and 79% for the more stable iron-bound form [143]) and thereafter enter the intestine from where it is absorbed into the circulation, but liposomalization or encapsulation has been shown to enhance availability and effect [144,145]. It is also important to note that digestion with enzymes in the GI tract (pepsin, trypsin or chymotrypsin) yields lactoferrin fragments that are still able to bind iron [146], and that fragments of lactoferrin have antimicrobial activity [39], which may be stronger than that of the intact protein [59,60]. Peptides of lactoferrin are considered promising antivirals, but isolation costs and stability pose challenges to reach the clinical phase [42], thus, at present the whole intact protein or food products/supplements with high content of lactoferrin are more accessible. A hindrance for use in medicine is the classification of lactoferrin products (as well as egg white powder and lysozyme discussed below) as food supplements, where these are not intended to treat disease, there is no controlled system for reporting effectiveness, and the active ingredient is not always of the same quality or integrity [142].

Ovotransferrin is abundant in hen egg white (12 g/l egg white) [147]. Methods to pasteurize egg whites use temperatures that minimize damage to heat sensitive proteins in the egg white, such as lysozyme and ovotransferrin [148–150]. Dried egg white powder, where 250 egg whites are equivalent to 1 kg powder, sold as a supplement is a compressed source of these proteins. Although 0.5–2.5% of children have an allergy to egg white, about 70% outgrow the allergy – nevertheless, many medicines and vaccines have ingredients derived from egg [151]. Iron-bound ovotransferrin is more resistant to gastrointestinal digestion [152,153], with iron-bound ovotransferrin well absorbed after ingestion [154]. Ovotransferrin is more readily digested by pepsin in the stomach compared with lysozyme [155]; however, the bioactive peptides (antimicrobial, antioxidant, anti-inflammatory and ACE-inhibitory) of ovotransferrin, as well as other egg white proteins, described earlier have mostly been produced by digestion that simulates that occurring in the GI tract [156], and these peptides resist further digestion [157] and are readily transported into human intestinal cells [158]. Ovotransferrin does however lose iron-binding activity after hydrolysis [147]. Simpler protocols with better yield and purity as well as low cost, will enable the use of isolated egg white proteins such as ovotransferrin or their peptides as pharmaceuticals [79], while presently the most readily available source is egg white powder.

Egg white is also the most readily available source of lysozyme (3.8 g/l egg white) [147]. Isolated HEWL is commercially available and is labeled as a food supplement by the European Commission. It is however available as lysozyme hydrochloride tablets (10, 30 or 90 mg), granules (10 or 20%) and syrup (0.5%) in Japan, and is prescribed by doctors to improve expectoration in bronchitis, bronchial asthma and bronchiectasis [159]. It is also sold as a food or pharmaceutical grade powder, and is widely used in the food industry as well as in pharmaceutical products (e.g., eye drops, wound healing creams, oral health products and over-the-counter drugs). Widerspread use is hindered somewhat by the isolation cost ($2.05/g) and there is significant effort being made around the world for commercial production of lysozyme, especially human lysozyme [160,161]. Methods to produce human lysozyme, which has higher enzymatic activity than HEWL and is therefore preferred, are under development (e.g., using transgenic rice), although these have a higher production cost than for HEWL [160]. However, the antiviral and immunomodulatory effects do not derive from the enzymatic activity, and the most available form of lysozyme currently is HEWL, whether isolated or in egg white. Oral administration of HEWL results in systemic effects – after oral administration of 90–900 mg HEWL in human subjects peak plasma concentrations are reached within an hour (with overnight fasting increasing absorption by sevenfold) and return to undetectable levels after 2 days [159,162]. While HEWL is fairly resistant to digestion in the stomach and partially resistant to digestion in the duodenum [155,163], enzymatic hydrolysis does produce antimicrobial fragments and broadens the antimicrobial spectrum [164].

Susceptibility of proteins to proteolytic digestion is very strongly related to protein stability [165], and polyols or their derivatives are commonly used to enhance protein stability in formulations [166]. An easily accessible and safe polyol may therefore be considered to improve stability of lactoferrin, ovotransferrin and HEWL following ingestion, and here it is suggested that glycerol may be a particularly suitable supportive solvent for the powdered sources of lactoferrin, ovotransferrin and HEWL. Glycerol is a low cost, readily available, sweet-tasting polyol, with excellent solvent and emulsifying properties, which is safe for ingestion and widely used in pharmaceutical applications (such as cough syrups) [167–169]. It is known to effectively stabilize proteins as well as refold denatured proteins [170,171], thereby restoring activities of enzymes that were inactivated by diverse processes [170]. In particular, glycerol was already shown to protect ovotransferrin and lysozyme when these proteins were subjected to stresses [150,166], and to partially restore the structure/activities of these proteins after denaturation [150,172]. Other properties may add further benefit, including anti-inflammatory [173] and antiviral effects [174,175], as well as the ability to inhibit ACE activity and decrease angiotensin II [176].

## Conclusion & future perspective

In view of the direct antiviral effects of lactoferrin, ovotransferrin and lysozyme against a wide range of viruses (including SARS-CoV-2 for lactoferrin) and their antimicrobial effects against a wide range of bacteria and fungi that may cause secondary infections in COVID-19 patients [177]; their immunomodulatory properties which stimulate antimicrobial responses yet promote resolution of inflammation, and in particular their previously shown beneficial effects in counteracting pathological neutrophil infiltration, macrophage activation, free iron overload, oxidative stress, AGE effects, excessive proinflammatory cytokine production (IL-6 and TNF-α in particular), and thrombus formation, which all feature in severe COVID-19; and their abundance and good safety profile – further testing of their potential role in prevention of SARS-CoV-2 infection or prevention of severe COVID-19 is suggested. The main suggestion is to use these antimicrobials upon presentation of symptoms to prevent noncritical cases from progressing to critical cases, although they may also be considered as a preventative for those at high risk of infection where lower quantities could be taken as a means of lowering risk of infection. Since the number of SARS-CoV-2 infection cases is growing so rapidly, the most expedient way to achieve this is through oral administration, which is suitable in the case of lactoferrin, ovotransferrin and lysozyme as these substances have systemic effects following ingestion. It is further suggested that, in the current circumstances of the COVID-19 pandemic, good quality nonisolated forms of these (such as egg white powder, bovine colostrum powder and other nonultrahigh temperature milk products with appreciable lactoferrin content) should also be tested while ensuring the desired concentrations of each antimicrobial are met, especially in settings where the isolated forms may not be as readily accessible. In favor of this suggestion, studies using lactoferrin-containing milk or lactoferrin-supplemented yoghurt have shown clinical efficacy in viral diseases [107,178], pasteurized whole milk has shown the effect of switching macrophages from M1 to M2 [97], several peptides in milk are antithrombotic [71], and several peptides in egg white besides those in ovotransferrin show supportive antioxidant as well as ACE-inhibitory effects [79,111]. However, in those individuals who are already critically ill and on ventilators, more care may need to be taken with the approach. Here, perhaps lactoferrin and lysozyme could be considered for intravenous administration or nebulization – a liposomal bovine lactoferrin nebulizer product is available. The accessibility and reasonable cost (in comparison to some of the other drugs – such as remdesivir and tocilizumab – under investigation to treat COVID-19) make these antimicrobials attractive as a therapeutic option and we therefore call for their rapid testing in clinical trials.

**Executive summary**

**Pathology of COVID-19: the role of free iron & oxidative stress in tissue injury**

- Severe COVID-19 is reminiscent of hyperferritinemic syndrome.
- Ferritin contributes to the inflammation by directly activating macrophages, and free iron may be liberated from ferritin by free radicals.
- Free iron reacts with oxygen or hydrogen peroxide to form free radicals, thereby driving oxidative stress and leading to tissue injury.

**Lactoferrin, ovotransferrin & lysozyme as potential therapeutics in COVID-19**

- There is no established effective treatment for COVID-19, and some treatment options being explored are unlikely to be widely available soon, especially in resource-limited settings.
- An abundant and safe antimicrobial that could act via oral ingestion to lower the risk of infection or prevent mild cases from progressing to severe disease would be ideal.
- Tear lactoferrin and lysozyme levels predict the risk of acquiring upper respiratory tract infections, and these antimicrobials are abundant in natural secretions.
- Ovotransferrin is more abundant than lactoferrin and can substitute lactoferrin in many applications.

**Lactoferrin as an antiviral & immune modulator**

- Bovine lactoferrin has been shown to inhibit SARS-CoV and SARS-CoV-2 cell entry.
- Lactoferrin restores iron homeostasis through sequestering free iron and modulating levels of proteins involved in controlling iron balance between blood and tissues.
- Lactoferrin reduces oxidative stress and inflammation, and it is immune ‘sensing’ with its effect dependent on the environment.
- Oral administration of lactoferrin in animal models and human studies of viral diseases, as well as various inflammatory disease states, shows beneficial effects and safety.

**Ovotransferrin as an antiviral & immune modulator**

- Ovotransferrin has antiviral peptides that are conserved with those found in human and bovine lactoferrin, and ovotransferrin may have a more potent antiviral effect.
- Ovotransferrin has immunomodulatory, antioxidant, anti-inflammatory and angiotensin-converting enzyme-inhibitory activities.

**Lysozyme as an antiviral & immune modulator**

- Lysozyme exhibits antiviral activity via its cationic peptides and has immunomodulatory, antioxidant and angiotensin-converting enzyme-inhibitory properties.
- Oral administration of lysozyme in animal models and human studies shows its ability to limit inflammation systemically, resulting in decreased immune-driven pathology.

**Sources, forms & practical use of lactoferrin, ovotransferrin & lysozyme**

- Lactoferrin is abundant in cow’s milk, while ovotransferrin and lysozyme are abundant in hen egg white.
- High costs of isolation have limited widerspread use of purified forms of these antimicrobials.
- Isolation costs and stability pose challenges for bioactive peptides of these antimicrobials to reach the clinical phase.

**Future perspective**

- These antimicrobials could be used upon presentation of symptoms to prevent noncritical cases from progressing to critical cases, and lower quantities could be taken to lower risk of infection in those at high risk.
- Good quality nonisolated forms of these should also be tested while ensuring the desired concentrations of each antimicrobial are met, especially in settings where the isolated forms may not be as readily accessible.
- The accessibility and reasonable cost make these antimicrobials attractive as a therapeutic option and we therefore call for their rapid testing in clinical trials.
